# Relationship between clinically assessed heart failure severity and the Tei index in Nigerian patients

**DOI:** 10.1186/1756-0500-6-488

**Published:** 2013-11-26

**Authors:** Olarinde Jeffrey Ogunmola, Anthony Olubunmi Akintomide, Adeyemi Michael Olamoyegun

**Affiliations:** 1 Cardiac Centre, Department of Internal Medicine, Federal Medical Centre, P.M.B. 201, Ido Ekiti, Ekiti State, Nigeria; 2 Department of Medicine, Cardiology Unit, Obafemi Awolowo University Teaching Hospitals Complex, P.M.B. 5538, Ile-Ife, Osun State, Nigeria; 3 Endocrinology, Diabetes and Metabolism Unit, Department of Internal Medicine, Ladoke-Akintola University of Technology Teaching Hospital, P.M.B. 4000, Ogbomoso, Oyo State, Nigeria

**Keywords:** Tei index, Heart failure, New York Heart Association, Systolic function, Diastolic function

## Abstract

**Background:**

The Tei index is a Doppler-derived myocardial performance index. It is a measure of the combined systolic and diastolic myocardial performance of both the left and right ventricles. The incidence of heart failure (HF) is increasing globally, and its severity can be clinically assessed using the New York Heart Association (NYHA) functional classification and more objectively using echocardiographic assessment of systolic and diastolic functions. Thus, a measure of the combined systolic and diastolic myocardial performance could be a useful predictor of the severity of the clinical status of patients with HF.

**Results:**

Seventy-five newly presenting patients with HF of NYHA class II to IV and 60 normal controls were consecutively recruited. Using conventional two-dimensional and Doppler echocardiography techniques, the left ventricular parameters assessed were the isovolumic relaxation time (IVRT), isovolumic contraction time (IVCT), ejection time (ET), ejection fraction (EF), and end-diastolic volume (EDV). The Tei index was determined using the formula IVCT + IVRT/ET. The mean Tei index of patients was significantly higher than that of controls (0.884 ± 0.321 vs. 0.842 ± 0.14; p < 0.001). The Tei index ranged from 0.33 to 1.94 in patients and from 0.56 to 1.24 in controls. The mean EF was lower in patients than in controls (50.47% ± 19.01% vs. 68.35% ± 7.75%; p = 0.001). The mean EDV was higher in patients than in controls (171.39 ± 100.96 vs. 94.15 ± 28.54; p < 0.001). Comparison of the mean Tei indices of patients with HF of NYHA classes II, III, and IV showed statistically significant differences among all three groups (p < 0.001).

**Conclusions:**

The Tei index seems to be a clinically relevant indicator of cardiac function. It is reflective of the severity of HF as clinically assessed using the NYHA functional classification in patients with HF.

## Background

Heart failure (HF) is one of the most common diseases of the heart in adults and accounts for an increasing number of hospital admissions in the developed world [1]. In Africa, at least 7% to 10% of all hospital admissions are a result of HF [2,3]. It has been demonstrated that systolic and diastolic dysfunctions coexist in the majority of patients with congestive HF [4-7]. Echocardiography has become the noninvasive method of choice for the assessment of either systolic or diastolic left ventricular (LV) function [8], and echocardiographic findings are known to influence management decisions and outcomes [9].

The ideal test for HF would be a noninvasive, integrated assessment of systolic and diastolic LV function that does not artificially uncouple systolic from diastolic function, is independent of ventricular loading conditions, and is reproducible during serial follow-up. A new Doppler-derived index combining systolic and diastolic time intervals proposed by Tei et al. in 1995 [10,11] fulfills some of these criteria. The Tei index has proven to be a reliable method for the evaluation of LV systolic and diastolic performance, with clear advantages over older established indices. It takes into account both systolic and diastolic function and is reportedly simple, reproducible, and independent of heart rate [12-14], blood pressure [11,13,14], and sex [15] while not appearing to be significantly affected by loading conditions [16]. The index is not influenced by LV geometry; hence, it does not change significantly with enlargement or remodeling of the heart [14,17,18]. The Tei index is defined as the sum of the isovolumic contraction time (IVCT) and isovolumic relaxation time (IVRT) divided by the ejection time (ET). It has already been clinically applied in patients with various etiologies of HF [10,11,19]. It is useful in the diagnosis of mild to moderate HF [13] and can reasonably separate normal subjects from those with HF [13,19].

The aim of the present study was to assess the relationship between the Tei index and HF of different severities clinically assessed using the New York Heart Association (NYHA) functional classification in patients with HF.

## Methods

### Study population

Seventy-five consecutive patients who fulfilled the Framingham criteria [20] for diagnosis of HF were recruited for this study. For the control group, 60 apparently healthy volunteers (without HF) were also consecutively recruited. The inclusion criteria were adult Nigerians 18 years and older with symptomatic HF who gave informed consent to participate in the study. The following patients were excluded: those younger than 18 years, those who did not partake in or complete the investigative profiles for the purpose of this study, those with poor echocardiographic windows or poor-quality echocardiograms, those not in sinus rhythm or on paced rhythm, those with a bundle branch block or high-grade atrioventricular block, and those with severe mitral regurgitation [21-23].

### Study location

This study was conducted at the Cardiac Care Unit, Department of Medicine, Obafemi Awolowo University Teaching Hospitals Complex, Ile-Ife, Osun State, Nigeria.

### Study design

This was a case–control study.

### Ethical consideration

Approval was obtained from the ethics and research committee of the hospital, and signed informed consent was obtained from each patient who participated in the study. The study protocol conformed to the ethical guidelines of the 1975 Declaration of Helsinki as reflected in *a priori* approval from the institution’s human research committee.

### Sample size estimation

The minimum sample size was calculated using a formula for estimating proportions with populations of less than 10,000: nf = n/1 + n/N. The value nf is the desired sample size when the population is less than 10,000; N is the estimated population size (this was estimated as the average of 150 new patients with HF seen annually in the cardiac unit); n is obtained using the formula n = z^2^pq / d^2^, where z is the standard normal deviate using a 95% confidence level of 1.96; p is the proportion of the target population estimated to have a particular characteristic (the prevalence of HF is 2.8%–16%^21^; therefore, the midpoint is 9.4%); q is obtained using the formula 1.0 – p; and d is the degree of accuracy desired, set at 0.05.

Thus,

$$n={\left(1.96\right)}^{2}\times 0.094\times 0.906/{\left(0.05\right)}^{2}=130.87,$$

and

nf=130.87/1+130.87/150=70.

### Clinical assessment

The diagnosis of HF was made using the Framingham criteria [20,24] for definitive HF. NYHA functional classes were determined on admission. Anthropometric measurements included height, weight, waist circumference, and body mass index. Peripheral arterial pulses were assessed and blood pressures were measured on admission [25,26].

### Imaging procedures

For each patient, a chest radiograph (posteroanterior view) was obtained at the radiology department to assess the cardiac silhouette, aorta, and lung fields. A conventional resting 12-lead electrocardiogram was obtained with a Schiller AT-2 electrocardiographic machine. Lead II was recorded for a long rhythm strip. The recommendations of the American Heart Association [27] concerning standardization of leads and instrument specifications were followed.

### Echocardiographic examination

Two-dimensional (2-D), motion mode (M-mode), and Doppler studies were performed with transthoracic echocardiography using a Siemens Sonoline G60S ultrasound imaging system with a P4-2 transducer. Measurements were performed in accordance with the recommendations of the American Society of Echocardiography [28] with leading-edge-to-leading-edge recordings taken. Calculations were made using the internal analysis software of the echocardiographic device. The M-mode measurement of LV functions was performed using the Teichholz formula. The 2-D views were used for real-time evaluation of morphological characteristics and as a reference for the selection of the M-mode beam. The echocardiographic views utilized for the study included the parasternal long-axis, short-axis, apical four-chamber, and apical five-chamber views. Pulsed Doppler echocardiographic recordings of the mitral inflow were obtained from the apical four-chamber view to assess LV filling dynamics. The Tei index was calculated from the ratio of time intervals expressed by the formula a – b / b or IVCT + IVRT / ET [29]. Measurements were taken from three consecutive beats and averaged. The parameters for the formula were determined by first locating the sample volume at the tips of the mitral valve leaflets in the apical four-chamber view, which enabled the measurement of ‘a’ (the time interval between the end and the start of transmitral flow). The sample volume was then located in the LV outflow tract, just below the aortic valve (apical five-chamber view) for the measurement of ‘b’ (LV ejection time). The interval ‘a’ included the IVCT, ET, and IVRT.

### Data analysis

The data were analyzed with a statistical computer software package (SPSS version 16.0). Continuous variables are expressed as mean ± standard deviation, and categorical variables are expressed as percentages. Student’s t-test was used to determine the difference between two means. A p-value of ≤0.05 was considered statistically significant.

## Results

The sample frame comprised age- and gender-matched patient and control groups (p = 0.62 and p = 0.55 for age and gender, respectively), as shown in Table 1. The patient group comprised 42 (56.0%) male and 33 (44.0%) female patients, while the control group comprised 38 (63.3%) male and 22 (36.7%) female subjects. The mean ages of the patients and controls were 56 ± 18.33 (range, 22–88) and 57 ± 18.46 (range, 23–88) years, respectively (p = 0.753). There was no statistically significant difference in age between the two groups (p = 0.069). The EF was lower in patients with HF than in controls (50.47% ± 19.01% vs. 68.37% ± 7.79%), and the difference was statistically significant (p = 0.001). The Tei index was easily obtained in all study subjects. The index was significantly higher (p < 0.001) in patients with HF than in control subjects (Table 1). The Tei index ranged from 0.33 to 1.94 in patients and from 0.56 to 1.24 in controls. Patients with HF received combination therapy comprising diuretics, ACE inhibitors, and aspirin or warfarin. The etiology of HF in the study population is shown in Table 2; the majority of HF was due to systemic hypertension (68.0%). As also shown in Table 2, patients with preserved systolic function had a lower Tei index than did patients with reduced systolic function, and the difference was statistically significant (p = 0.003). As shown in Table 3, there were significant differences in the mean EF among the NYHA classes (p < 0.001). The mean EF diminished from NYHA classes II to IV. In addition, significant differences were present in the mean end-diastolic volumes among NYHA classes II, III, and IV (see Table 3). The mean end-diastolic volume was higher in NYHA classes II to IV (p < 0.001). A total of 41% of patients with HF recruited for this study presented in NYHA class IV, and the others presented in NYHA classes III (30.6%) and II (28%). Comparison of the mean Tei indices in NYHA classes II, III, and IV by means of one-way analysis of variance showed statistically significant differences among all three groups (p < 0.001). According to Tukey’s post hoc test, the group comparisons that showed statistically significant differences were those between NYHA classes II and IV (p < 0.001) and III and IV (p < 0.001); no statistically significant differences were observed between II and III (p = 0.487). As shown in Table 4, the EF and fractional shortening showed significantly negative correlations, while the other hemodynamic parameters showed positive correlations with the Tei index.

**Table 1:** Demographic parameters, mean ejection fraction, and Tei index of patients and matched controls

**Variables**	**Patients (n = 75) n (%)**	**Controls (n = 60) n (%)**	**p value**
**Age (years)**			
18–44	9 (12.0)	6 (10)	0.62
45–64	25 (33.6)	21 (34.6)	
≥65	41 (54.4)	33 (55.4)	
**Gender**			
Male	42 (56.0)	38 (63.3)	0.55
Female	33 (44.0)	22 (36.7)	
**Age (years)**^**1**^	56 ± 18.33	57 ± 18.46	0.0753
**Ejection fraction**^**1**^	50.47 ± 19.01	68.37 ± 7.79	0.001
**Tei index**^**1**^	0.88 ± 32	0.36 ± 0.07	<0.001

^1^Mean ± standard deviation.

**Table 2:** Etiology and ejection fraction of patients with heart failure

**Variables**	**Patients (n = 75)**
**Etiology**	n (%)
Systemic hypertension	51 (68.0)
Dilated cardiomyopathy	10 (13.3)
Rheumatic heart disease	9 (12.0)
Congenital heart disease	2 (2.7)
Endomyocardial fibrosis	2 (2.7)
Acute myocardial infarction	1 (1.3)
**Mean EF versus TI of patients (t = 3.03, p = 0.003)**	
EF ≤ 45% (n = 32)	0.970 ± 0.34
EF > 45% (n = 43)	0.799 ± 0.27

EF, ejection fraction; TI, Tei index.

**Table 3:** Left ventricular function assessment across NYHA classes

**Variables**	**NYHA II**	**NYHA III**	**NYHA IV**	**p value**
Mean ejection fraction^1^	65.48 ± 16.38	53.69 ± 15.59	37.92 ± 14.35	<0.001
Mean end-diastolic volume^1^	91.50 ± 62.77	160.33 ± 76.42	233.73 ± 98.27	<0.001
Mean Tei index^1^	0.59 ± 0.14	0.79 ± 0.12	1.15 ± 0.30	<0.001

^1^Mean ± standard deviation; NYHA, New York Heart Association.

**Table 4:** Correlation of conventional echocardiographic hemodynamic indices with the derived Tei index in the study population

**Variable**	**Correlation (r)**	**p**
EF	0.353	0.001
LVDD	0.570	0.001
LVSD	0.640	<0.0001
LVEDV	0.350	<0.010
Mitral E/A ratio	0.238	0.022
DT	0.220	0.032

EF, ejection fraction; FS, fractional shortening; LVDD, left ventricular diastolic dimension; LVSD, left ventricular systolic dimension; LVEDV, left ventricular end-diastolic volume; DT, deceleration time.

## Discussion

The Tei index has been proven to be a reliable method for the evaluation of LV systolic and diastolic performance, with clear advantages over older established indices and prognostic value in many kinds of heart disease [10,13,14,18,30]. Our study revealed a relationship between clinically assessed HF severity and the Tei index. This relationship in a black African population between a bedside clinical marker of severity (NYHA classification) and the Tei index further confirmed the usefulness of the Tei index in assessing hemodynamic functions of patients with HF. The Tei index in patients with HF was higher and had a wider range than that in normal controls. This higher Tei index in patients than in healthy individuals was due to prolongation of the isovolumic time intervals and a shortening of the ET. Similar findings were reported by Bruch et al. [13] and Dujardin et al. [31].

Moreover, the mean Tei index was higher with increased HF severity, and there were statistically significant differences among all three groups (NYHA II, III, and IV). This finding is in agreement with several others, including the findings obtained by Tei et al. [10]. Their study included patients with HF of NYHA class II to IV with EFs of 30% to 50%, and a statistically significant difference in the mean Tei index was present among the NYHA functional groups. In addition, Ohno et al. [32] showed that patients with advanced NYHA classifications or patients with restrictive LV filling patterns (which reflect higher pulmonary wedge pressure) and advanced congestive HF also exhibited an increased myocardial performance index (Tei index).

Our study also revealed the trend of presentation of patients with HF in our environment (41% of these patients were in NYHA class IV). In most such patients, the HF is structurally advanced with an altered LV geometric pattern, and the ellipsoid shape of the heart tends to become spherical, leading to limitations in the use of most of the traditional parameters for assessing systolic and diastolic functions. This further buttresses the relevance of the Tei index. The present study also demonstrated the usefulness of the Tei index in HF of causes other than hypertensive HF, as seen in a study of black patients in Africa [33]. The dilated cardiomyopathies found in this study were diagnosed based on clinical and classic echocardiographic findings. None were found to have resulted from ischemia, alcohol, or any specific cause within our limited resources. Another important finding in this study was the significant correlation between the Tei index and other conventional indices of systolic and diastolic functions.

Furthermore, in agreement with previous studies such as that by Bruch et al. and Ambakederemo et al. [13,19], the Tei index in controls was helpful in differentiating between normal controls and patients with mild to moderate HF (NYHA II and III functional classification). It was also useful in differentiating patients with severe HF (NYHA IV) from normal controls. More studies of African individuals are needed to determine the cut-off Tei index in normal subjects that would clearly separate them from patients with HF.

The herein-described myocardial performance index (Tei index), which combines systolic and diastolic time intervals as an expression of global myocardial performance, correlates with cardiac function and seems to be a useful complimentary marker in the assessment of LV function. This combined measurement of ventricular chamber performance may be more reflective of overall cardiac dysfunction than systolic or diastolic measures alone. In our environment, where people present in the late stage of severe clinical HF, the Tei index may be more effective because it is not based on a geometric model or on volume measurement. A study of healthy adults and patients with congestive HF reported by Correale et al. [34] investigated the clinical agreement between the Tei index measured conventionally and that measured by pulsed-wave tissue Doppler of the mitral annulus. Both methods had similarly high diagnostic accuracy for cardiac HF; however, this report addressed the use of a higher myocardial performance index cut-off point for the best diagnostic accuracy when using the new pulsed-wave tissue Doppler index method [35].

This study has some limitations. First, the small sample size did not allow for adequate stratification for the observation of the presence or absence of statistical significance. Second, the effects of loading conditions and arrhythmias on this index remain to be elucidated. Finally, pseudonormalization of the index value was present in this study.

## Conclusions

In conclusion, this study indicates that the Tei index is a useful myocardial performance index that has a promising role in determining overall cardiac dysfunction. It is a reliable indicator of clinically assessed severe HF using the NYHA functional classification.

## Competing interests

The authors declare that they have no competing interests.

## Authors’ contributions

OJO designed the study, was involved in the data collection and statistical analysis, wrote the protocol, and was involved in writing the first through final drafts of the manuscript. AAO was involved in designing the study and in writing the first through final drafts of the manuscript. OMA was involved in the writing of the manuscript. All authors read and approved the final manuscript.
